# What should we focus on in pregnancy complicated by pheochromocytoma? a bibliometric analysis (1990-2024)

**DOI:** 10.3389/fonc.2025.1557376

**Published:** 2025-07-17

**Authors:** Shiyun Deng, Fan Li, Jing Tian, Congcong Sun, Yanqing Zhang

**Affiliations:** ^1^ Department of Anesthesiology, University-Town Hospital of Chongqing Medical University, Chongqing Medical University, Chongqing, China; ^2^ Department of Gynecology and Obstetrics, University-Town Hospital of Chongqing Medical University, Chongqing Medical University, Chongqing, China

**Keywords:** pheochromocytoma, pregnancy, bibliometric, CiteSpace, paraganglioma

## Abstract

**Purpose of review:**

The incidence of pheochromocytoma and paraganglioma (PPGL) in pregnancy is extremely low, yet it poses a significant threat to maternal and fetal safety. While studies are published annually, progress in this field remains slow. A key question is how researchers can better utilize limited case data to gain valuable insights. This bibliometric review summarizes the current research landscape, highlights recent findings, and suggests areas for future investigations.

**Methods:**

A comprehensive search was conducted in the Web of Science Core Collection (WoSCC), PubMed, and Embase databases for PPGL in pregnancy from 1990 to 2024. Data analysis was performed using Excel 2022 and CiteSpace.

**Current state:**

A total of 391 articles were included in the analysis. The United States was the most prolific country, and the Mayo Clinic was the most productive institution. Lenders JWM was identified as both the most published and most co-cited author. *Journal of the Endocrine Society* was the most frequently targeted journal for publication, while the *Journal of Clinical Endocrinology & Metabolism* had the highest number of co-citations. Evidence-based practice in this field primarily depends on case reports, case series, case-control studies, and systematic reviews. The primary focus in this research area is on clinical management and pregnancy complications.

**Recent findings:**

Maternal and infant mortality in pheochromocytoma during pregnancy has significantly decreased due to improved awareness and advances in diagnosis and treatment. Antepartum diagnosis is the most vital element in reducing mortality; hypertension at admission and history of PPGL were independent factors of antepartum diagnosis. Abdominal/pelvic tumor location and catecholamine levels ≥10 times the upper limit of the reference range were associated with adverse outcomes. Women with hereditary disease and risk of developing PPGL should be screened before becoming pregnant for occult PPGL and should be treated adequately.

**Conclusion:**

Enhanced collaboration between countries and institutions is needed to advance the field. Diagnostic and therapeutic strategies, as well as complications associated with PPGL during pregnancy, have consistently been core areas of research. Future studies should prioritize the clarification of detailed clinical management protocols and the underlying pathophysiological mechanisms, with the goal of generating high-quality evidence to guide the care of this high-risk population.

## 1 Introduction

Pheochromocytoma and paraganglioma (PPGL) are rare neuroendocrine tumors. Pheochromocytomas originate in the adrenal medulla, while paragangliomas arise from extra-adrenal chromaffin tissue. Due to their similar histological features and clinical behavior, they are collectively referred to as PPGL (1). PPGL is a rare cause of secondary hypertension in pregnancy, with an extremely low incidence but it poses significant risks to both maternal and fetal health. The reported mortality rates for mothers and infants have reached as high as 48% and 54%, respectively (2). Although international perioperative guidelines for PPGL have been published, there are currently no specialized guidelines for cases involving pregnancy. Clinically, the symptoms of pregnancy-associated PPGL closely resemble those of pregnancy-induced hypertension, which often leads to misdiagnosis and delayed treatment. Diagnosis after delivery is widely recognized as a critical risk factor that increases mortality and complications for both mothers and fetuses (3–7). In fact, the diagnosis of this condition itself is not particularly difficult; the primary challenge is considering PPGL as a potential diagnosis when treating a pregnant patient (8). Therefore, increased awareness of PPGL in pregnancy is essential.

Almost every year, case reports of PPGL in pregnancy are published in medical databases, underscoring its rarity. The earliest report dates back to 1949, and to date, case studies remain an essential source of evidence-based knowledge for managing pregnant patients with PPGL. Significant advancements have been made in many areas of endocrine research; however, progress in studying PPGL in pregnancy remains slow. The scarcity of cases makes it challenging to obtain high-quality evidence-based data for this condition (8). After searching the Cochrane Library, we found no relevant randomized controlled trials. The current evidence in evidence-based medicine comes mainly from case reports, systematic reviews. A key issue in the current context is how to better utilize sporadic clinical cases to obtain more valuable insights. Herein, we summarize existing literature on PPGL in pregnancy to create an up-to-date knowledge map and identify future research directions.

Bibliometrics is a quantitative method used to analyze publication trends and research output within a specific field. CiteSpace, one of the most widely used tools for bibliometric analysis, was developed by Professor Chaomei Chen (9). It enables researchers to identify influential authors, institutions, countries, references and keywords, as well as to analyze citation patterns and emerging trends. Bibliometric methods have been widely applied in oncology research to explore the current landscape and evolving directions of the field. However, to date, no bibliometric analysis has focused specifically on PPGL in pregnancy. The present study aims to perform a bibliometric analysis of research on PPGL during pregnancy, with the goal of summarizing recent developments and identifying major research hotspots in the field.

## 2 Materials and methods

### 2.1 Data source

Three researchers independently searched the Web of Science Core Collection (WoSCC), PubMed, and Embase databases for articles related to pregnancy complicated by PPGL, covering the period from 1990 to 2024.

### 2.2 Search strategy

The search strategies for PubMed and Embase were based on a combination of Medical Subject Headings (MeSH) or Emtree terms and free-text keywords. Controlled vocabulary terms included “Pregnancy,” “Pheochromocytoma,” and “Paraganglioma,” while the free-text terms consisted of synonyms and variations for each concept. The search strategy for WoSCC involved the use of keywords appearing in the title and abstract of the articles. Details of the search strategy can be found in the **Supplementary Material**.

### 2.3 Data collection

The inclusion criteria were as follows: (1) the content of the article, or specific sections thereof, pertains to pregnant women with PPGL, regardless of whether the diagnosis was made antepartum or postpartum; patients with PPGL during pregnancy who have other comorbidities were also included; (2) article types limited to original research articles and reviews; (3) publications written in English; and (4) publication period spanning from 1990 to 2024.

The exclusion criteria were as follows: (1) studies unrelated to the topic, such as those involving non-pregnant patients with PPGL or pregnancy-related hypertension not caused by PPGL; (2) non-journal publications, such as books, conference abstracts, proceedings, and letters; and (3) studies involving non-human subjects.

### 2.4 Data analysis

**Figure 1:**
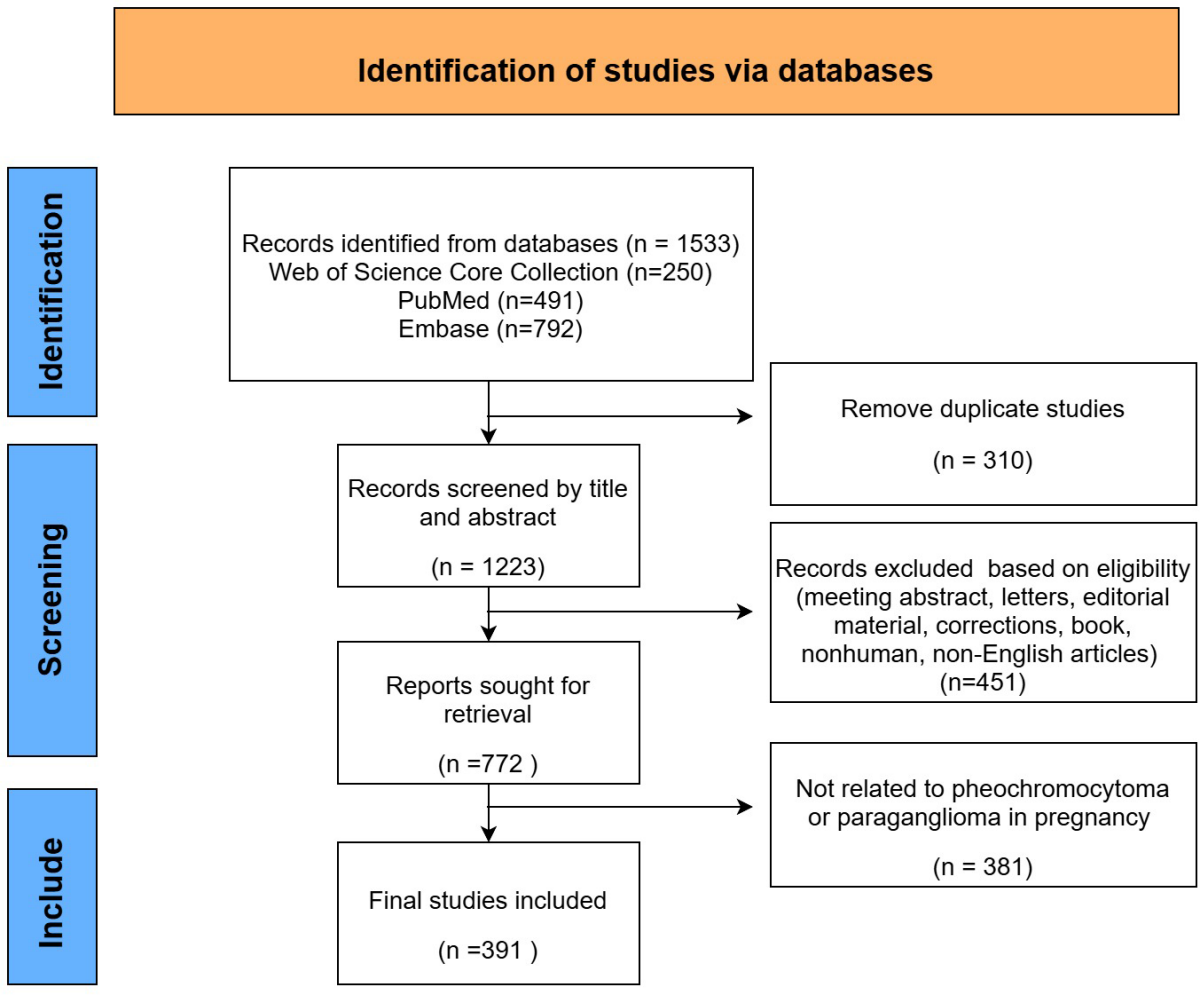
Data collection and filtering flow diagram.

After data collection and filtering (**Figure 1**), 391 articles were imported into Microsoft Office Excel 2022, and a publication volume chart was created.

Epidemiological characteristics of pregnancies complicated by PPGL were extracted from the included literature, including prevalence, maternal and fetal mortality rates, as well as the rates of antepartum and postpartum diagnosis.

Bibliometric analysis was performed using CiteSpace. Key indicators analyzed included publishing countries, institutions, authors, co-cited authors, co-cited journals, references, and keywords. The evaluation metrics included publication count, co-citation frequency, betweenness centrality, burst strength, and JCR journal rankings. Co-citation frequency reflects how often two documents or authors are cited together in subsequent publications. A higher co-citation frequency indicates a stronger intellectual linkage or shared relevance between the cited entities within the field. Betweenness centrality quantifies the extent to which a node lies on the shortest paths between other nodes, indicating its role as a bridge or connector within the network. A betweenness centrality score greater than 0.1 is typically considered indicative of a node with significant bridging influence in the network. ​Citation burst strength reflects the intensity of a sharp increase in citations of a particular reference over a specific period. A higher burst strength indicates that the reference received considerable attention in a short time, suggesting its impact on emerging research frontiers. The clustering analysis of major keywords employed the Log-Likelihood Ratio (LLR) algorithm. The modularity Q and weighted average silhouette S were calculated to assess clustering effectiveness. The Q value ranges from 0 to 1, reflecting the network structure’s quality. A Q value greater than 0.3 indicates a significant clustering structure. The S value ranges from -1 to 1, positively correlating with the clustering network’s rationality. An S value greater than 0.5 indicates a reasonably structured network, while an S value greater than 0.7 suggests reliable clustering results. JCR classifications were obtained from Journal Citation Reports 2023.

## 3 Results

### 3.1 Global trend of publications and epidemiological overview

#### 3.1.1 Annual number of publications

**Figure 2:**
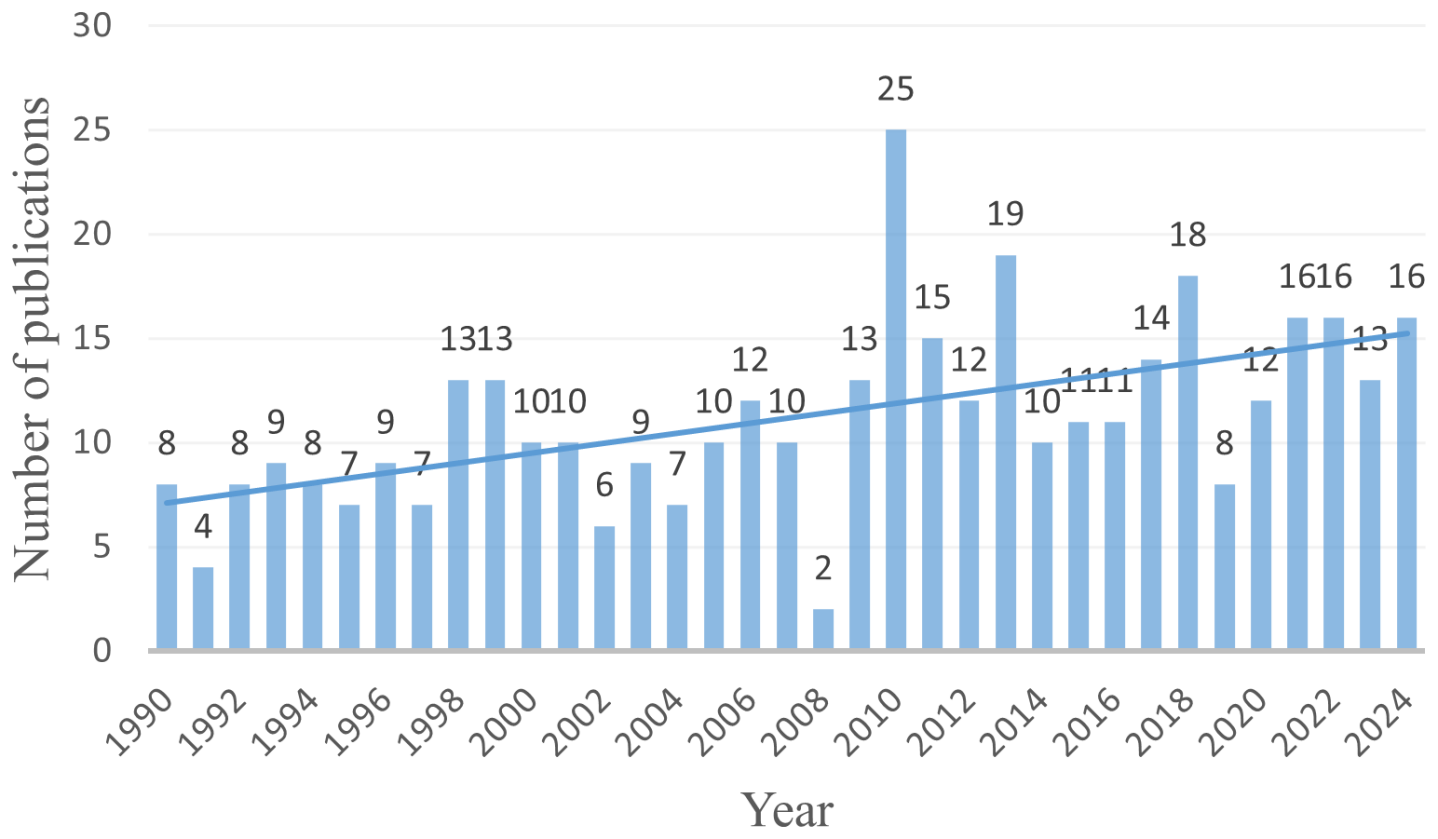
Annual output of research of pregnancy complicated by PPGL.

Based on the search strategy, a total of 391 articles related to pregnancy complicated by PPGL were identified from January 1, 1990, to December 30, 2024. As shown in **Figure 2**, the annual number of publications has shown a steady upward trend, reflecting growing academic interest in PPGL during pregnancy.

#### 3.1.2 Country and institution contributions analysis

**Table 1** presents the top 10 countries and institutions ranked by the number of publications, along with their corresponding publication counts and betweenness centrality scores. The countries with the highest number of publications were the United States (n = 61, 15.6%), Italy (n = 19, 4.8%), Japan (n = 17, 4.3%), and India (n = 17, 4.3%). The countries with the highest centrality were the United States (0.21) and Germany (0.14), while all other countries had a centrality score of 0. The leading institutions in terms of publication volume were the Mayo Clinic (USA, n = 4), King Edward Memorial Hospital (India, n = 4), and the Chinese Academy of Medical Sciences (China, n = 3). All institutions had a centrality score of 0. A centrality score of zero indicates that the country or institution did not function as a bridge within the collaboration network, suggesting limited connectivity or a relatively isolated research position.

**Table 1:** The top 10 countries and organizations by publication volume.

Rank	Country	Publications	Centrality	Organization	Publications	Centrality
1	USA	61	0.21	Mayo Clinic (USA)	4	0
2	Italy	19	0	King Edward Memorial Hospital (India)	4	0
3	Japan	17	0	Chinese Academy of Medical Sciences (China)	3	0
4	India	17	0	Carl Gustav Carus University (Germany)	3	0
5	China	15	0	Northwestern University Medical School (USA)	3	0
6	France	15	0	Chulalongkorn University (Thailand)	3	0
7	Netherlands	12	0	Technische Universitat Dresde (Germany)	3	0
8	Canada	12	0	Hopital Universitaire Europeen Georges-Pompidou (France)	2	0
9	Australia	11	0	Radboud University Nijmegen (Netherlands)	2	0
10	Germany	9	0.14	University of Florence (Italy)	2	0

#### 3.1.3 Authors and co-cited authors analysis

The top 10 authors by publication count and co-citation frequency are listed in **Table 2**. The most prolific authors were Lenders JWM (n = 5), Menon PS (n = 4), and Shah NS (n = 5). The top three co-cited authors were Lenders JWM (co-citation count = 125, centrality = 0.27), Harper MA (co-citation count = 71, centrality = 0.38), and Schenker JG (co-citation count = 38, centrality = 0.27). Notably, Lenders JWM from Radboud University Nijmegen in the Netherlands was both the most prolific and most co-cited author, with a centrality score of 0.27, indicating his pivotal position within the co-citation network.

**Table 2:** The top 10 authors and co-cited authors in research on pregnancy complicated by PPGL.

Rank	Author	Documents	Co-cited author	Co-citation	Centrality
1	Lenders JWM	5	Lenders JWM	125	0.27
2	Menon PS	4	Harper MA	71	0.38
3	Shah NS	4	Schenker JG	38	0.27
4	Bandgar TR	3	Biggar MA	37	0.27
5	Eisenhofer G	3	Ahlawat SK	36	0.29
6	Sarathi V	3	Harrington JL	25	0.22
7	Mannelli M	3	Oliva R	25	0.06
8	Bancos I	2	Mannelli M	24	0.03
9	Buranasupkajorn P	2	Neumann HPH	12	0.12
10	Chudek J	2	Manger WM	12	0.02

#### 3.1.4 Journals and co-cited journals analysis

Journal publication counts were analyzed using EndNote, and **Table 3** displays the top 10 journals ranked by publication volume and co-citation frequency. The top three journals by publication volume were *Journal of the Endocrine Society* (n = 15, Q2), *Gynecological Endocrinology* (n = 9, Q2), and *Endocrinology and Metabolism Clinics of North America* (n = 5, Q1). Among the top 10 journals publishing on this topic, five were classified as endocrinology journals, four as obstetrics and gynecology journals, and one was indexed in both categories. Co-cited journals were analyzed using CiteSpace, and eight of the top 10 co-cited journals were classified as Q1 journals according to the JCR. The three most co-cited journals were *The Journal of Clinical Endocrinology & Metabolism* (co-citation count = 66, Q1, centrality = 0.26), *European Journal of Endocrinology* (co-citation count = 55, Q1, centrality = 0.12), and *Obstetrical & Gynecological Survey* (co-citation count = 44, Q1, centrality = 0.10).

**Table 3:** The top 10 journals and co-cited journals for research of PPGL in pregnancy.

Rank	Journal	Documents	JCR	Co-cited Journal	Co-citation	JCR	Centrality
1	Journal of the Endocrine Society	15	Q2	The Journal of Clinical Endocrinology & Metabolism	66	Q1	0.26
2	Gynecological Endocrinology	9	Q2	European Journal of Endocrinology	55	Q1	0.12
3	Endocrinology and Metabolism Clinics of North America	5	Q1	Obstetrical & Gynecological Survey	44	Q1	0.10
4	Cureus	5	Q3	British Journal of Surgery	36	Q1	0.14
5	Endocrine	4	Q2	The New England Journal of Medicine	35	Q1	0.11
6	Journal of Obstetrics and Gynecology Research	4	Q3	BJOG: An International Journal of Obstetrics & Gynecology	35	Q1	0.14
7	Journal of Endocrinological Investigation	4	Q2	Obstetrics & Gynecology	31	Q1	0.06
8	Journal of Obstetrics and Gynecology	4	Q4	Journal of Endocrinological Investigation	29	Q2	0.06
9	Obstetrics & Gynecology	4	Q1	Australian & New Zealand Journal of Obstetrics & Gynecology	28	Q3	0.08
10	Revista Española de Anestesiología y Reanimación	4	Q3	Hypertension	28	Q1	0.11

#### 3.1.5 Epidemiological trends

The incidence rate of pregnancy-associated PPGL is generally low, estimated to be 0.000351% to 0.006612% (10–12), as shown in **Table 4**.

**Table 4:** The incidence of PPGL associated with pregnancy.

Incidence rate	Cases/Samples	Sample source	Period	Country	Article
0.006612%	2/30246	Mayo Clinic	1975-1996	United States	Adrenal tumors and pregnancy
0.000478%	21/4390869	Multiple mandatory national health registries in Sweden	1973-2015	Sweden	Maternal pheochromocytoma and childbirth in Sweden 1973–2015: a population-based study on short and long-term outcome
0.000351%	10/2852099	UK Obstetric Surveillance System	2011-2015	United Kingdom	Hormone-secreting adrenal tumors cause severe hypertension and high rates of poor pregnancy outcome; a UKOSS study with case control comparisons

According to retrospective studies and systematic reviews, maternal and fetal mortality rates have markedly improved in recent years compared to earlier periods (3). Antepartum diagnosis remains the most critical factor in reducing both maternal and fetal mortality (3–6, 13), with hypertension at admission and a history of PPGL identified as independent predictors of timely diagnosis (6). Initiation of alpha-adrenergic blockade after diagnosis effectively controls symptoms caused by catecholamine excess and reduces the risk of adverse outcomes (3, 14). Tumors located in the abdomen or pelvis, and plasma catecholamine levels exceeding 10 times the upper limit of the reference range, are both associated with poorer outcomes (3). Maternal mortality is also higher among patients who experience hypertensive crises compared to those who do not (7).

Six studies reported outcomes for pregnant women and fetuses, as detailed in **Table 5**. Among them, Bancos I et al. (3), published in 2021 in *The Lancet Diabetes & Endocrinology*, conducted the longest retrospective study spanning from 1980 to 2019, including 232 cases and a total of 249 pregnancies, showed a maternal mortality rate of 1% and a fetal mortality rate of 9% for pregnancies associated with PPGL. Although the mortality rate has significantly decreased, 6.5% of mothers experienced severe cardiac complications related to catecholamine excess. Other systematic reviews covered different periods: 1988-1997 (15); 1998-2008 (14); 1980-2019 (3); 1988-2019 (6); 2000-2011 (7); and 2010-2017 (13), reporting maternal and fetal mortality rates respectively, as shown in **Table 5**. These rates are significantly reduced compared to the 48% maternal mortality rate reported before 1979 (2).

**Table 5:** Diagnosis rate, maternal and fetal mortality according to the time of diagnosis, as reported in the literature for different periods.

Results Periods		1988-1997	1998-2008	1980-2019	1988-2019	2000-2011	2010-2017
Diagnosis Rate	No. of pregnancies	42	60	249	204	77	63
	Postpartum (%)	16.7	30.0	31.3	29.4	27.3	38.0
	Ante-partum (%)	83.3	70.0	68.7	70.6	72.7	62.0
Maternal Mortality	Overall (%)	4.8	12.0	1.3	9.0	7.8	6.3
	Postpartum diagnosis (%)	14.3	28.0	3.9	29.3	28.6	16.7
	Ante-partum diagnosis (%)	2.9	5.0	0.0	0.7	0.0	0.0
Fetal Mortality	Overall (%)	11.9	17.0	8.7	14.2	16.7	11.1
	Postpartum diagnosis (%)	0.0	28.0	13.0	25.0	28.6	16.7
	Ante-partum diagnosis (%)	14.3	12.0	6.5	9.7	12.3	7.7

All data in the table are derived from their respective literature sources. Diagnosis rate are calculated based on pregnancy frequencies. Antepartum diagnosis includes diagnoses made before or during pregnancy, while postpartum diagnosis includes diagnoses made after delivery or upon autopsy. Maternal and fetal/neonatal mortality rates are calculated separately, using the total number of pregnant women and the total number of fetuses/neonates as their respective denominators.

### 3.2 Effects of hormones on mothers and fetuses

**Figure 3:**
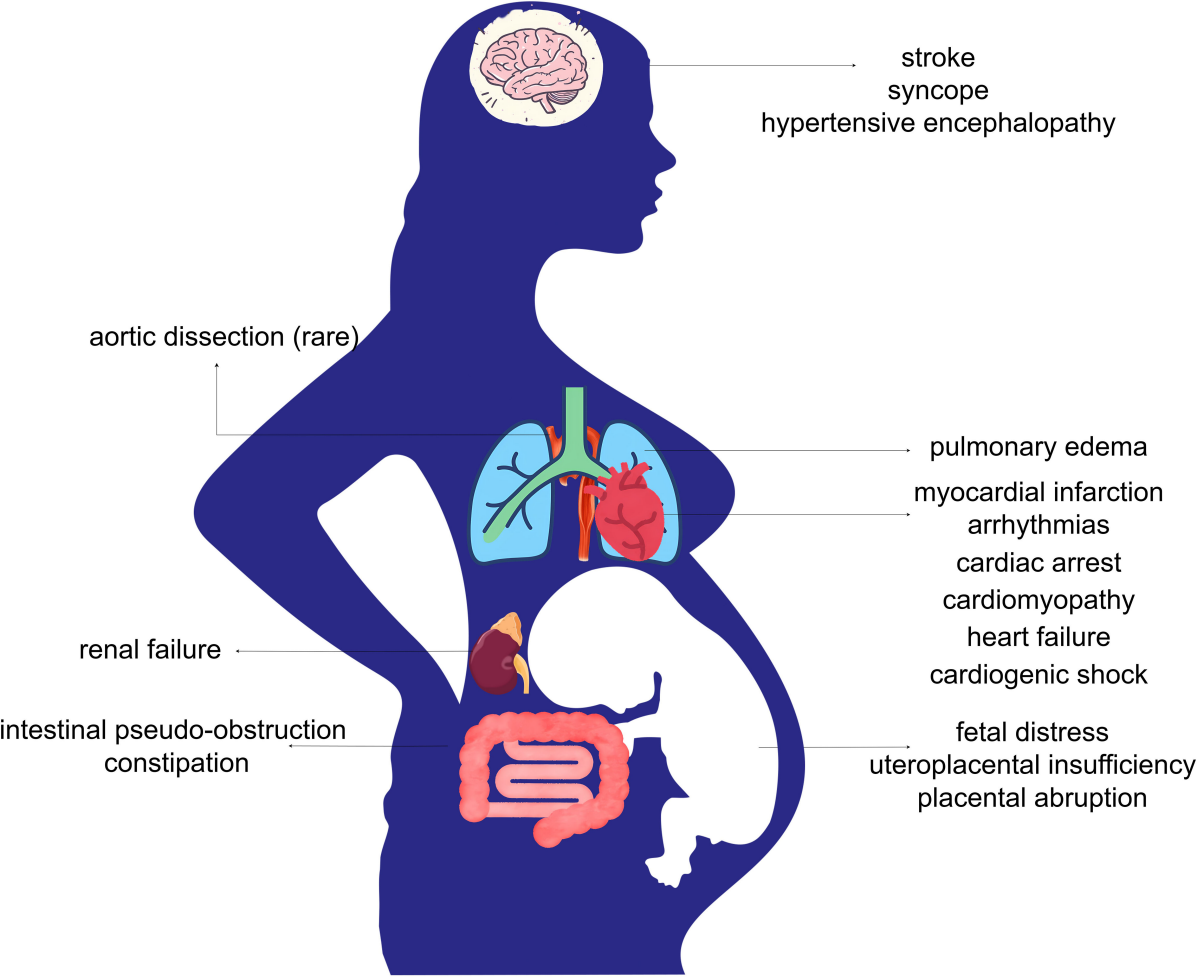
Complications of functional PPGL in pregnancy. Excessive secretion of catecholamines in functional PPGL leads to systemic vasoconstriction, heightened sympathetic activity, hemodynamic instability, and increased cardiac workload. These pathophysiological changes may result in serious maternal complications, including cardiovascular and cerebrovascular events, pulmonary edema, renal failure, and gastrointestinal dysfunction. Fetal complications may arise from impaired placental perfusion, leading to intrauterine fetal distress, uteroplacental insufficiency, and placental abruption.

**Figure 3** summarizes the impact of excessive catecholamine secretion on both the mother and the fetus as reported in case reports.

Excessive catecholamines exert differing effects on mothers and fetuses. In the mother, elevated catecholamine levels cause widespread vascular spasm, intense myocardial contraction, and a hypermetabolic state, leading to potential damage across multiple organ systems that are directly exposed. However, for the fetus, the risks associated with PPGL are primarily linked to maternal placental vasoconstriction (13). The placental barrier protects the fetus from direct exposure to high maternal catecholamine levels. Research evidence shows that the concentrations of norepinephrine, epinephrine, and metanephrine in umbilical cord blood are lower than those in maternal plasma (16, 17). Therefore, placental vasospasm-induced hypoxia, placental insufficiency, and placental abruption represent the primary threats to the fetus (18). The uteroplacental circulation lacks autoregulation it is directly affected by changes in maternal blood flow, indicating that effective control of maternal blood pressure is beneficial for positive fetal health outcomes (13, 16).

Following the diagnosis of PPGL, 3% of pregnant women opted for elective termination, while 4% experienced miscarriage or intrauterine fetal loss between 8 and 37 weeks of gestation (3). Preterm delivery occurred in 26%–30% of cases (3, 11), with some reports indicating rates as high as 45% (6). However, the incidence of small for gestational age (SGA) infants did not significantly differ from that in the general obstetric population, possibly due to timely termination or appropriate perinatal management. The incidence of infant respiratory distress syndrome (IRDS) was 13% among neonates born to mothers with PPGL, substantially higher than the 1.8% observed in the general population (11). In the long term, no significant difference in hospitalization rates was observed between children born to mothers with PPGL and those born to healthy mothers, based on a median follow-up period of 12 years (11).

### 3.3 Citation analysis and keyword analysis

#### 3.3.1 Co-citation analysis and citation burst analysis

The top 10 co-cited references and their bibliographic details are listed in **Table 6**. Highly co-cited references represent foundational literature that has shaped the development of this research field. The top three citations are as follows: in 2013, Biggar MA et al. published a paper titled “Systematic review of pheochromocytoma in pregnancy” in the *British Journal of Surgery*. In 2010, Oliva R et al. published a paper titled “Pheochromocytoma in Pregnancy: A Case Series and Review” in *Hypertension*. In 2012, Lenders JWM et al. published a paper titled “Pheochromocytoma and pregnancy: a deceptive connection” in the *European Journal of Endocrinology*.

**Table 6:** The top 10 most co-cited publications in research on pregnancy complicated by PPGL.

Rank	Title	Author	Journal	JCR	Publication year	Co-citation
1	Systematic review of phaeochromocytoma in pregnancy	Biggar, MA	British Journal of Surgery	Q1	2013	38
2	Pheochromocytoma in pregnancy: A case series and review	Oliva R	Hypertension	Q1	2010	35
3	Pheochromocytoma and pregnancy: a deceptive connection	Lenders JWM	European Journal of Endocrinology	Q1	2012	43
4	Pheochromocytoma and pregnancy	Lenders JWM	Endocrinology and Metabolism Clinics of North America	Q1	2019	13
5	Maternal and fetal outcomes in phaeochromocytoma and pregnancy: a multicenter retrospective cohort study and systematic review of literature	Bancos I	The Lancet Diabetes & Endocrinology	Q1	2021	10
6	Endocrinology in pregnancy: Pheochromocytoma in pregnancy: case series and review of literature	Van der Weerd K	European Journal of Endocrinology	Q1	2017	9
7	Pheochromocytoma in pregnancy: When is operative intervention indicated?	Junglee N	Journal of Women’s Health	Q1	2007	7
8	Adrenal tumors and pregnancy	Harrington JL	World Journal of Surgery	Q2	1999	6
9	Pregnancy and phaeochromocytoma/paraganglioma: clinical clues affecting diagnosis and outcome - a systematic review	Langton K	BJOG: An international journal of obstetrics & gynecology	Q1	2021	5
10	Phaeochromocytoma in pregnancy	Grodski S	Internal Medicine Journal	Q2	2006	5

**Figure 4:**
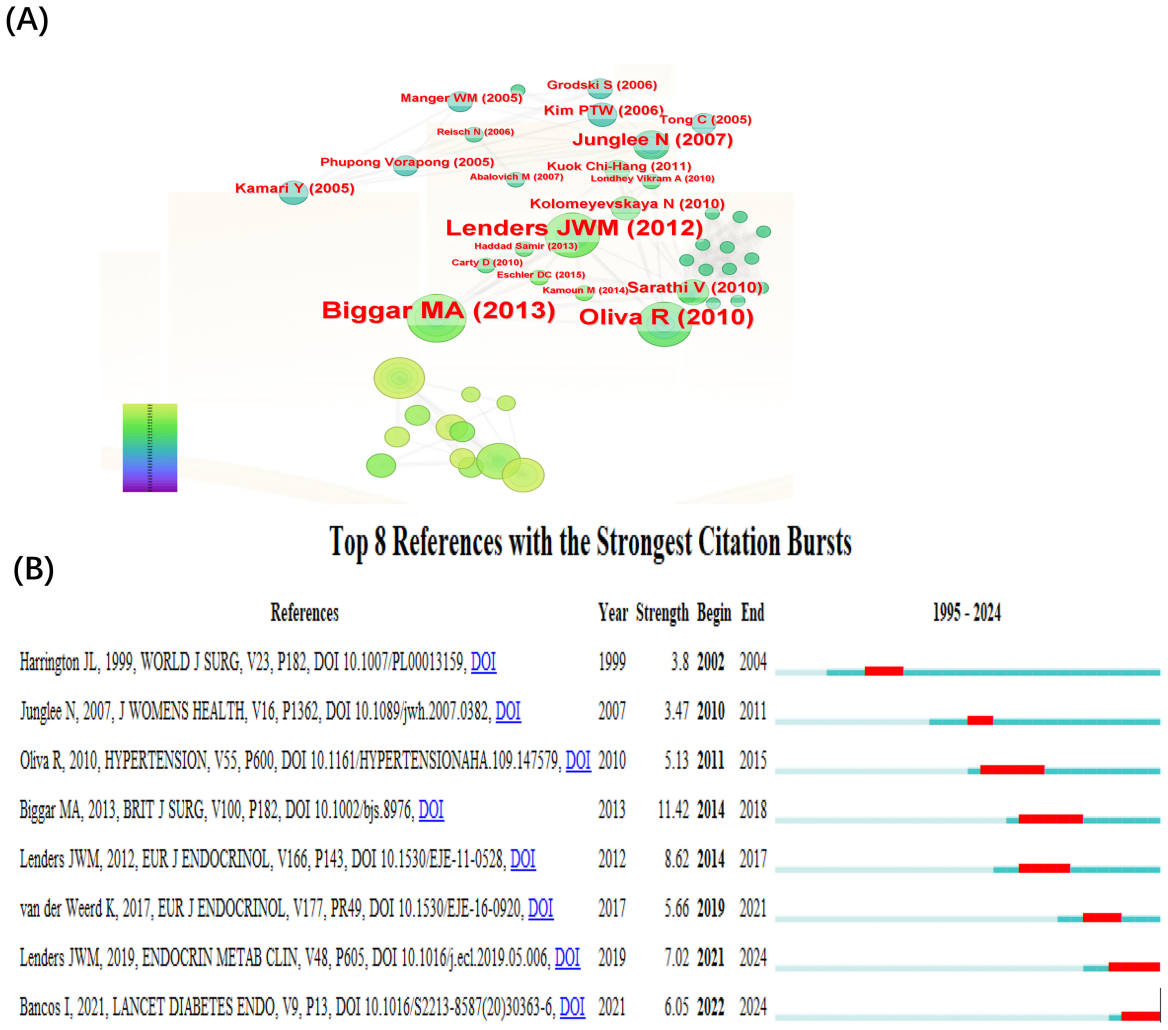
Co-citation reference visualization and citation burst. **(A)** Co-cited literature interaction. **(B)** Reference burst analysis.

**Figure 4A** displays the co-citation reference network generated by CiteSpace. Each node represents a cited reference, with larger nodes indicating higher co-citation frequency. The color of the nodes reflects the publication year, showing the temporal distribution of influential literature. Purple outer rings highlight references with high betweenness centrality, signifying their bridging role across different thematic areas. Edges between nodes indicate co-citation links, where thicker lines reflect stronger intellectual connections between references. **Figure 4B** illustrates the top 8 references with the strongest citation bursts identified by CiteSpace. The red segments indicate the period during which each reference experienced a significant surge in citations, while the blue lines represent the overall timespan from 1995 to 2024. The burst strength reflects the intensity of the citation increase within a specific period. Among these, the article by Biggar MA (2013) in the *British Journal of Surgery* exhibited the highest burst strength (strength=11.42, from 2014 to 2018), suggesting that it received a rapid and concentrated increase in scholarly attention during that time. Since 2021, citation bursts have been observed for the articles by Lenders JWM (2019), titled “Pheochromocytoma and Pregnancy” in *Endocrinology and Metabolism Clinics of North America*, and Bancos I (2021), “Maternal and fetal outcomes in phaeochromocytoma and pregnancy: a multicenter retrospective cohort study and systematic review of literature,” published in *The Lancet Diabetes & Endocrinology*, indicating a recent surge of academic interest in clinical management strategies and outcome-based research on PPGL in pregnancy.

#### 3.3.2 Keyword analysis

**Figure 5:**
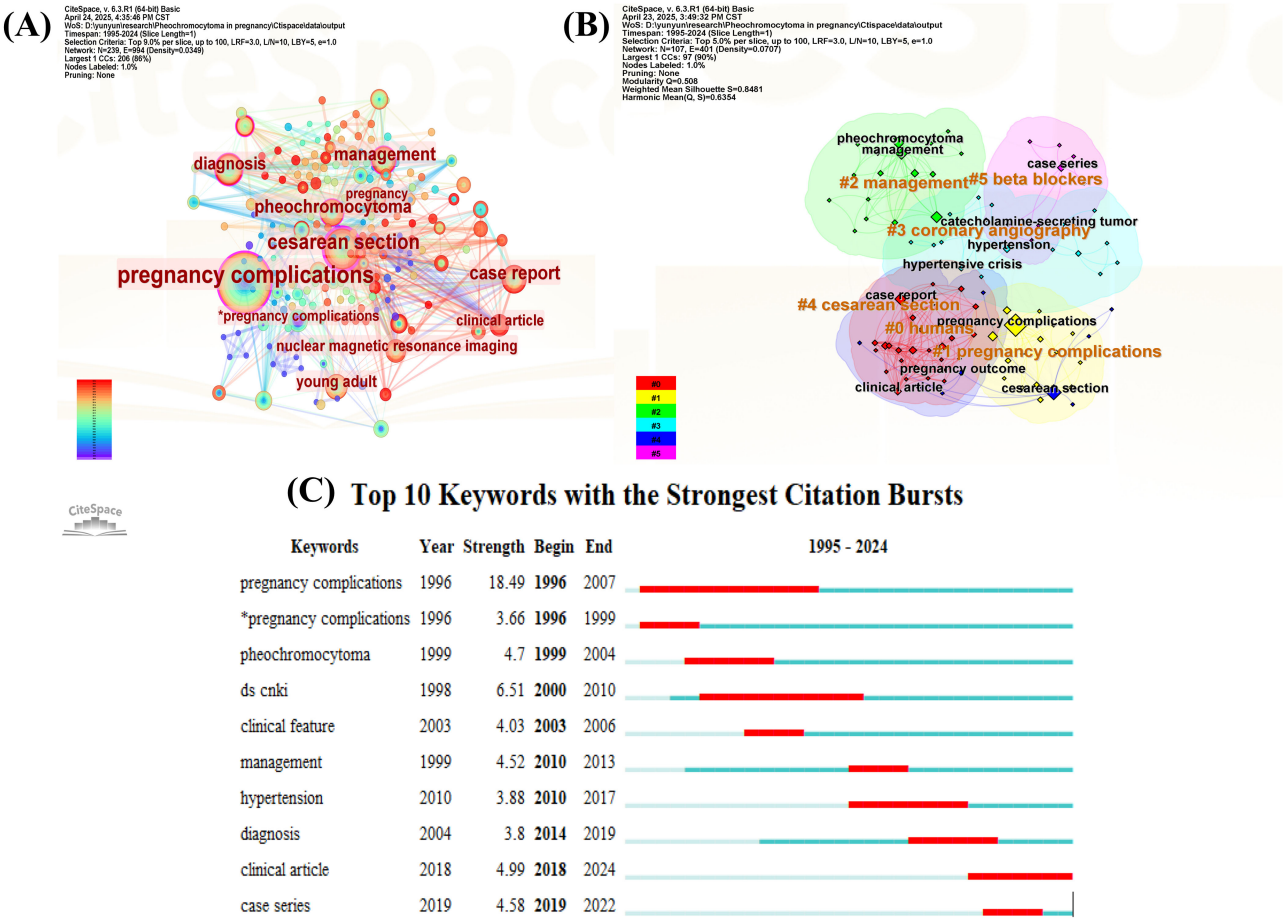
Visual network of keywords in pregnancy complicated by PPGL. **(A)** Keywords visualization map of the CiteSpace network. **(B)** Cluster diagram of keywords co-occurrence analysis network. **(C)** Keywords burst analysis.

A keyword co-occurrence network was visualized using CiteSpace, with the g-index set to k=25 to enhance the clarity of keyword distribution. The top 10 keywords include pregnancy complications (n = 128, centrality = 0.21), cesarean section (n = 60, centrality = 0.59), management (n = 34, centrality = 0.15), case report (n = 34, centrality = 0.08), pheochromocytoma (n = 33, centrality = 0.18), diagnosis (n = 26, centrality = 0.35), young adult (n=23, centrality = 0.20), nuclear magnetic resonance imaging (n=19, centrality = 0.02), clinical article (n=19, centrality = 0.08), and pregnancy (n=15, centrality = 0.07). **Figure 5** presents the visualized keyword network for studies on pregnancy complicated by PPGL from 1995 to 2024. **Figure 5A** illustrates the keyword co-occurrence network, with node colors ranging from purple (1995) to red (2024), representing temporal evolution. The size of each node reflects the frequency of keyword occurrences, the width of the connecting lines indicates the strength of co-occurrence, and the color of the lines represents the time of co-appearance. Nodes with a purple outer ring represent high burst strength. **Figure 5B** shows the results of keyword clustering analysis, with representative cluster labels including “ 0 humans”, “ 1 pregnancy complications”, “ 2 management”, “ 3 coronary angiography”, “ 4 cesarean section”, and “ 5 beta blockers”. These cluster labels classify keywords into distinct research themes; clusters with smaller label numbers contain a larger number of keywords. The degree of overlap among cluster labels reflects the thematic relevance and conceptual overlap between different research areas. The modularity Q score of 0.5 and silhouette S score of 0.84 indicate a stable and reasonable clustering structure. Burst analysis of keywords, as shown in **Figure 5C**, identified ten keywords with significant citation bursts. Among them, the most recent keywords with notable burst strengths were “clinical article” (burst strength = 4.99) and “case series” (burst strength = 4.58). The keyword with the strongest burst strength was “pregnancy complications” (burst strength = 18.49).

## 4 Discussion

### 4.1 General information

Research on pregnancy complicated by PPGL remains limited in quantity, with case reports being the predominant type of publication, followed by review articles. There is a lack of strong collaborative networks among publishing countries and institutions, and greater international and inter-institutional collaboration is encouraged in future research. Lenders JWM is a highly influential author in this field, with a primary research focus on the clinical management of pregnancy complicated by PPGL. Endocrinology and obstetrics and gynecology journals are the most preferred publication venues in this area of research.

### 4.2 Key considerations in the management of pregnancy complicated by PPGL

**Figure 6:**
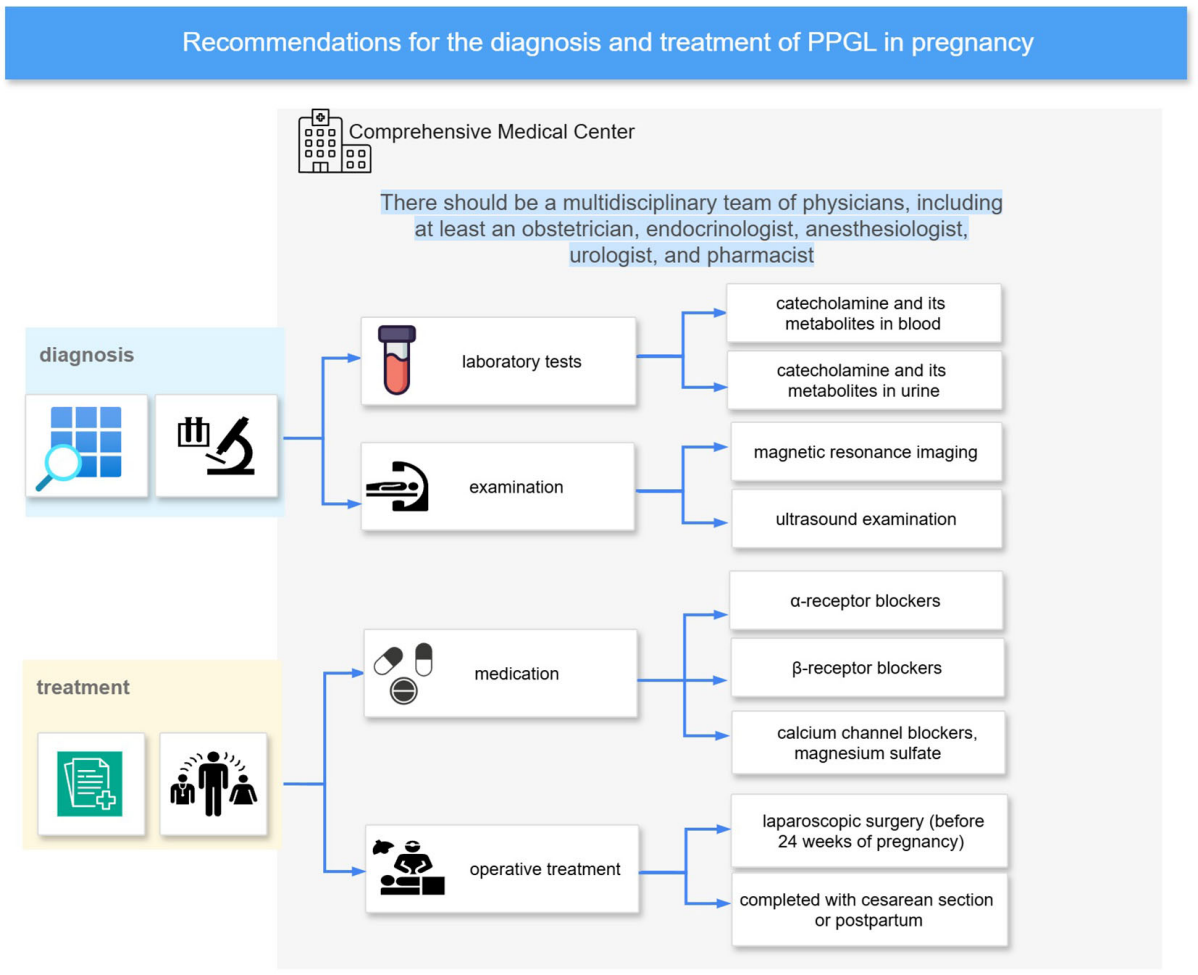
Diagnosis and treatment recommendations. It summarizes a recommended management pathway for PPGL in pregnancy, based on bibliometric analysis and key literature. The approach emphasizes care in Comprehensive Medical Center with multidisciplinary teams. Diagnostic evaluation includes catecholamine testing (blood and urine), MRI, and ultrasonography. Initial pharmacologic management typically involves α-blockers, with β-blockers and calcium channel blockers or magnesium sulfate added as needed.

Highly co-cited references and citation burst analyses across different time periods have revealed the foundational literature and evolving research hotspots in this field. Keyword co-occurrence and clustering analyses further illustrate the major themes and directions of research. Diagnosis and treatment remain central topics in the study of PPGL during pregnancy. As illustrated in **Figure 6**, prior studies have established a general consensus regarding multidisciplinary care, diagnostic strategies, and antihypertensive management. However, there are still many details in the clinical management strategies that need to be further investigated. These include the sensitivity and specificity of diagnostic tools, additional tests following diagnosis, target blood pressure levels, the necessity of fluid resuscitation, the role of the anesthesiologist, timing and mode of delivery, timing of tumor resection, medication-related precautions, and considerations for breastfeeding in the postpartum period. These key aspects will be discussed in detail in the following subsections.

#### 4.2.1 What is the sensitivity and specificity of the diagnostic methods?

Recommended diagnostic methods for pregnant patients suspected of having PPGL include hormone assays, ultrasound, and magnetic resonance imaging (MRI). MRI has a sensitivity of 90–100% for detecting adrenal tumors in late pregnancy, compared to 54% for ultrasound (14, 19). There is a lack of data regarding the specificity of biochemical testing in pregnant patients with PPGL. Generally, biochemical testing demonstrates relatively high specificity in diagnosing PPGL during pregnancy, as catecholamine and metabolite levels in plasma and urine are typically low in normal pregnancies (15), resulting in a low rate of false positives. When false positives do occur, they are commonly attributed to interference from medications such as methyldopa, labetalol, or tricyclic antidepressants, increased sympathetic activity, or improper sample collection. To minimize the risk of false-positive results, it is recommended that blood samples be obtained after the patient has fasted and remained in a supine resting position for at least 20 minutes (18, 20). In terms of diagnostic sensitivity, urine tests are superior to plasma tests: urinary catecholamines demonstrate a sensitivity of 93%, metanephrines 100%, plasma catecholamines 79%, and plasma metanephrines 100% (14).

Computed tomography (CT) offers the highest diagnostic sensitivity for PPGL; however, due to concerns about fetal radiation exposure, it is often used with caution in pregnant patients (21). In fact, studies have shown that fetal exposure to CT radiation in late pregnancy is well below the threshold considered to be harmful (22–25). At the same time, the potential risk of breast radiation exposure should also be considered when performing CT examinations on pregnant patients. Ionizing radiation is a known risk factor for breast cancer, and this risk is particularly relevant in younger women (26). Abdominal CT does not directly expose the breast to radiation; only a minimal amount of scattered radiation reaches the breast, resulting in a negligibly low dose (27). In contrast, chest CT examinations expose the breast to relatively high doses of radiation, with an average effective dose of approximately 7 mSv, depending on the scanning protocol and technical parameters (26). Therefore, radiologists should optimize scanning protocols to minimize breast radiation exposure while ensuring diagnostic quality. A retrospective cohort study based on health databases from Ontario, Canada (1995–2014), showed that thoracic CT or V/Q scan exposure during pregnancy or the early postpartum period was not associated with an increased short-term risk of breast cancer over a median follow-up of at least 5 years. (28). This result is consistent with the conclusion observed in the general population of women (26). However, evidence regarding long-term effects of chest CT exposure during pregnancy remains limited, and further studies are warranted to confirm these findings. Maternal mortality has been shown to increase significantly in the presence of hypertensive crises (7). Therefore, in cases of unexplained hypertensive emergencies or acute cardiovascular events during pregnancy, CT imaging may be appropriately considered by the attending physician to enable timely diagnosis and intervention (29).

#### 4.2.2 What are the additional tests after diagnosis?

Pregnant patients diagnosed with PPGL may have coexisting endocrine disorders, such as diabetes mellitus and excessive secretion of adrenal corticosteroids (30, 31). It is recommended to evaluate fasting blood glucose and serum cortisol levels following diagnosis. Fasting glucose testing is simple, widely available, and routinely performed during antenatal care. If the initial serum cortisol level is within the normal range, repeated testing is generally unnecessary; however, clinicians should remain alert to any signs or symptoms suggestive of hypercortisolism during pregnancy.

PPGL is well known for its highly variable clinical presentations. In severe cases, excessive catecholamine secretion can cause extensive cardiovascular and multi-organ damage. Reported complications include Takotsubo cardiomyopathy, myocardial infarction, pulmonary edema, cerebrovascular stroke, ischemic bowel obstruction, and acute renal failure (32). In pregnant patients with marked blood pressure fluctuations accompanied by symptoms such as palpitations, chest pain, nausea, vomiting, or dyspnea, clinical evaluation should include cardiac injury biomarkers, B-type natriuretic peptide (BNP), echocardiography, serum creatinine, and urinary microalbumin or albumin levels. Oliva R et al. pointed out in their review that proteinuria may serve as a useful marker to distinguish hypertension induced by pheochromocytoma from that of other pregnancy-related causes (33). However, a case series reported in China found that 3 out of 6 pregnant women with pheochromocytoma exhibited positive urinary albumin results (34). This may be attributed to sustained hypertension damaging the glomerular capillary walls, resulting in increased permeability and subsequent protein leakage into the urine. Therefore, proteinuria may also occur in pregnant patients with PPGL when blood pressure is poorly controlled.

#### 4.2.3 What is the goal of antihypertensive medication?

For functional PPGL, once the diagnosis is confirmed, α-blocker antihypertensive therapy is recommended. β-blockers are introduced only after the initiation of alpha-adrenergic blockade to counteract catecholamine-induced tachyarrhythmias and reflex tachycardia caused by alpha-adrenergic receptor inhibition. The most commonly used α-blockers are phenoxybenzamine and doxazosin (5, 35), and the β-blocker of choice is metoprolol (36). While drug selection and application have been extensively studied, fewer studies have addressed specific antihypertensive targets. ESC/ESH Guidelines for the management of arterial hypertension recommend a target of 140/90 mmHg for antihypertensive treatment during pregnancy (37). Van der Weerd et al. suggest in their literature review that for chronic hypertension without signs of end-organ damage, the target blood pressure during pregnancy should be < 150/100 mmHg with a diastolic pressure > 80 mmHg. If signs of end-organ damage are present, the target should be lowered to < 140/90 mmHg (5). Blood Pressure (BP) control strategies were last discussed in 2022, when Lucinda et al. proposed lower systolic BP targets of 90-110 mmHg and the heart rate should average less than 90 beats per minute (36). There is no evidence to support that less tight or tight BP control is better for pregnant women and fetuses. Keeping blood pressure at a stable value in pregnant patients with PPGL is challenging due to the enlarging uterus and fetal movements potentially compressing the tumor. Moreover, PPGLs express luteinizing hormone/chorionic gonadotropin (LHCG) receptors, and pregnancy may trigger catecholamine surges and hypertensive crises through hCG-mediated epinephrine stimulation (38). Although there are differences in antihypertensive goals, if BP is stabilized at the current dose and an increase in dose would result in orthostatic hypotension, it is recommended that the current dose be maintained (36, 39).

#### 4.2.4 Is rehydration therapy warranted?

Blood volume expansion through rehydration is a common protocol for perioperative preparation in patients with PPGL. However, during pregnancy, maternal blood volume increases by at least 30% compared to pre-pregnancy levels, peaking at 32–34 weeks. This may raise controversy regarding the necessity of rehydration therapy in managing PPGL during pregnancy. In the systematic review by Lucinda et al., they emphasize increasing fluid and sodium intake when starting an alpha-adrenergic blocker (36). Although pregnancy increases maternal blood volume, relative hypovolemia in PPGL is well recognized. Vasodilation and hypotension after alpha-blocker administration can further exacerbate volume deprivation; therefore, careful and balanced fluid management is advisable.

#### 4.2.5 What can anesthesiologists do to help?

Cesarean section is currently the primary mode of delivery (3, 6), which can be performed under intrathecal anesthesia, general anesthesia, or combined anesthesia techniques. In general, the preferred form of anesthesia for cesarean delivery is intrathecal anesthesia, which avoids the effects of general anesthesia drugs on the fetus. In uncomplicated parturients, general anesthesia is typically reserved for emergency situations or when neuraxial anesthesia is contraindicated (40). It is recommended that women with catecholamine levels exceeding 10 times the upper limit of normal and tumors located in the pelvic-abdominal cavity be administered general anesthesia during cesarean section. For these patients, general anesthesia can provide better circulatory control than intrathecal anesthesia and allows for a timely response to malignant hypertension and refractory hypotension. The standard general anesthesia regimen is propofol, rocuronium, and remifentanil for induction, with inhalation anesthetics for maintenance. The concentration of inhaled agents is kept low due to their uterine relaxation effects, which may compromise uterine tone. General anesthesia combined with intrathecal anesthesia can combine the advantages of both, guaranteeing good analgesia and reducing the dose of general anesthetic drugs, and is recommended for high-risk women with PPGL who are undergoing non-emergency surgery.

There are also cases where vaginal delivery has been safely performed with the involvement of an anesthesiologist (41–44). For women with indications for vaginal delivery, it is recommended that vaginal delivery be attempted with the participation of an anesthesiologist. Anesthesiologists can provide analgesia during labor, including intrathecal analgesia and intravenous analgesia, and effective analgesia can reduce the occurrence of malignant hypertension. Additionally, anesthesiologist can monitor and manage hypertensive crises with short-acting antihypertensive drugs.

#### 4.2.6 When should labor be induced?

In non-emergency situations—excluding conditions such as fetal distress or maternal life-threatening events—it is advisable to prolong pregnancy beyond 34 weeks if fetal growth and development are deemed normal, as neonatal survival improves with advancing gestational age. In China, the average survival rate for infants born before 28 weeks is 62.3% (45), increasing to 79.1% at 30 weeks and 85.8% at 31 weeks (46). Among the general obstetric population, antenatal corticosteroid administration has been shown to improve outcomes in preterm neonates (47). However, the use of corticosteroids for fetal lung maturation in pregnant patients with PPGL remains controversial. Some experts argue that corticosteroids may stimulate tumor secretion of dopamine, potentially triggering a pheochromocytoma crisis; therefore, their use is generally avoided in this population. Nevertheless, several case reports have documented successful corticosteroid administration in PPGL patients with threatened preterm labor (48–51). Moreover, since PPGL may coexist with Cushing’s syndrome (30, 52), maternal hypercortisolism should be excluded prior to corticosteroid initiation. Therefore, in non-emergency situations, antenatal corticosteroids may be cautiously administered in pregnant patients with PPGL at risk of preterm delivery before 34 weeks, provided that maternal blood pressure is closely monitored throughout the course of treatment.

#### 4.2.7 Cesarean section or vaginal delivery?

A Swedish population-based survey conducted between 1973 and 2015 reported that 57% of mothers with PPGL delivered vaginally (11). In contrast, most previous studies have indicated that the rate of cesarean section in patients with PPGL during pregnancy is generally higher than that of vaginal delivery (3–6, 14). This pattern likely reflects clinical concerns about the elevated risk of adverse outcomes associated with PPGL, which may influence decisions regarding the mode of delivery. However, current evidence suggests that the choice of delivery mode is not independently associated with maternal or neonatal outcomes (3, 11). In a systematic review by Langton et al., the maternal mortality rate was higher in vaginal deliveries (1 in 3) than in cesarean sections (1 in 12) (14); however, this conclusion was based on a limited sample size and requires confirmation through studies with larger cohorts.

#### 4.2.8 When to perform a tumor resection?

Surgical resection at the time of delivery or postpartum is the most commonly adopted strategy, accounting for approximately 70% of cases (3, 6, 11, 12). For patients diagnosed before 24 weeks of gestation with hormonally active PPGLs that are poorly controlled with medical therapy, antenatal tumor resection may be considered, typically between 14 and 23 weeks (5). Data suggest that patients who undergo antenatal tumor removal are more likely to achieve full-term gestation, and their neonates experience a lower incidence of low neonatal Apgar scores (defined by an Apgar score of <7) (6). Adverse maternal or fetal outcomes have been reported in approximately 3.8% (3/78) to 4.5% (4/88) of antenatal surgical cases, although it remains unclear whether antenatal surgery is conclusively associated with improved overall outcomes (3, 6). If surgical resection of the tumor is required, preoperative preparation with α-adrenergic blockade is essential (3, 18, 21).

#### 4.2.9 What are the precautions for drug use?

The use of beta-adrenergic blockers without prior alpha-adrenergic blockade is strictly contraindicated, as blocking peripheral beta-mediated vasodilation while unopposed alpha-adrenergic stimulation persists can result in a paradoxical elevation of blood pressure (53). Labetalol, a first-line agent for pregnancy-induced hypertension with an alpha-to-beta blockade ratio of approximately 1:3, is not recommended for use as monotherapy in pregnant patients with PPGL (53, 54). Phenoxybenzamine, a frequently used non-selective alpha-blocker, can cross the placenta and may cause neonatal hypotension and respiratory depression, with a reported fetal–maternal plasma accumulation ratio of approximately 1.6:1 (51, 55, 56). Doxazosin also crosses the placenta, but fetal plasma concentrations are lower than maternal levels, with a fetal-to-maternal concentration ratio of 0.8, indicating a relatively low risk of neonatal hypotension (17). To date, no cases of neonatal hypotension associated with doxazosin have been reported, and it is considered a safe alternative for use in pregnant patients with PPGL (5, 17, 36).

Medications that may stimulate catecholamine release from the tumor, trigger histamine release, or produce sympathomimetic effects should be avoided. These include opioid analgesics such as morphine and meperidine, antiemetics like metoclopramide, anesthetic agents such as thiopental and ketamine, neuromuscular blockers including mivacurium and succinylcholine, as well as sympathomimetic drugs such as ephedrine (54, 57, 58).

#### 4.2.10 Can postpartum mothers breastfeed?

Following tumor resection, plasma catecholamine and metabolite levels decrease rapidly (59). Patients who undergo tumor removal prior to delivery may breastfeed without restriction. In cases where cesarean section is combined with tumor resection, breastfeeding can be initiated once the final dose of medication has been sufficiently metabolized. For mothers requiring continued postpartum antihypertensive therapy without prior tumor removal, certain medications may be excreted into breast milk. Therefore, breastfeeding is generally not recommended or should only proceed under close clinical supervision. Limited evidence suggests that only 0.1% or less of doxazosin is transferred into breast milk (60), which is well below the commonly accepted 10% threshold for infant relative dose. However, this finding is based on isolated case reports and requires further validation. A case report by Aplin SC et al. (56) documented a mother–infant pair in which oral phenoxybenzamine was continued during breastfeeding without the occurrence of neonatal hypotension, suggesting a potential safety profile of phenoxybenzamine in lactating patients. Nevertheless, this conclusion is drawn from a single case and requires confirmation in larger cohorts.

### 4.3 Genetic studies on PPGL in pregnant patients

Patients carrying certain susceptibility genes have an increased risk of developing PPGL. Statistics show that approximately 40% of patients with PPGL have genetic mutations (61). The identified susceptibility genes for PPGL include NF1, RET, VHL, succinate dehydrogenase subunits (SDHA, SDHB, SDHC, SDHD), succinate dehydrogenase complex assembly factor (SDHAF2), TMEM127, MAX, EGLN1/PHD2, EGLN2/PHD1, KIF1β, IDH1, HIF2α, FH, and ATRX (35, 39, 62, 63). Mutations detected in pregnant patients include NF1 (64), RET (65–67), VHL, succinate dehydrogenase subunits (SDHA, SDHB, SDHC, SDHD) (3, 42, 68–70), MAX, FH, CDKN2B (3), and HIF2α (71). Associated endocrine disorders include multiple endocrine neoplasia type 2A (MEN2A) (65–67), von Hippel–Lindau (VHL) disease (72, 73), neurofibromatosis type 1 (NF1) (11, 64, 74–77), and Carney Triad (3).

Individuals with a past history of PPGL and/or heritable PPGL syndrome are considered high-risk and should undergo genetic counseling and tumor screening prior to conception (4, 14, 78), a process typically overseen by endocrinologists. Metastatic or invasive forms of PPGL are generally recognized as high-risk conditions, and maternal deaths have been reported in such cases (79). However, a large retrospective study by Bancos I et al. (3), which included 249 pregnancies complicated by PPGL, found that neither metastatic disease nor the presence of pathogenic germline mutations was associated with an increased risk of maternal or fetal complications or mortality. This investigation represents the largest cohort study to date on PPGL in pregnancy, and its findings challenge previous assumptions—possibly due to the exclusion of case reports and small case series with fewer than five patients, which may have introduced selection bias into earlier literature.

## 5 Limitations

Due to the rarity of PPGL in pregnancy, there is a lack of randomized controlled trials in this field. Large-scale cohort studies and case-control studies are also limited. Currently, the main body of evidence is derived from case reports, case series, systematic reviews, and a small number of observational studies. This concentration on lower levels of evidence may introduce publication bias and limit the overall strength and generalizability of the findings.

This study performed a bibliometric analysis of literature published between 1990 and 2024. Publications from 2025 were excluded due to insufficient time for citation accumulation. Additionally, non-English articles were excluded from the analysis, which may have led to the omission of relevant studies published in other languages.

## 6 Conclusion

This study represents the first bibliometric analysis specifically focusing on pregnancy complicated by PPGL. Through bibliometric and visual analytic methods, it highlights publication trends, current research landscapes, major hotspots, and emerging directions in this field. Future research should prioritize international collaboration, refine diagnostic and therapeutic strategies, and investigate the factors contributing to adverse maternal and fetal outcomes to provide more robust evidence-based support for clinical decision-making in this high-risk population.

## Data Availability

The original contributions presented in the study are included in the article/**Supplementary Material**. Further inquiries can be directed to the corresponding author.
