# Supplementary material for: What should we focus on in pregnancy complicated by pheochromocytoma? a bibliometric analysis (1990-2024)
Source: Front Oncol. 2025 Jul 17;15:1557376. doi: 10.3389/fonc.2025.1557376 (PMC12312014; doi:10.3389/fonc.2025.1557376)
Supplement: Supplementary file 1 [file DataSheet1.pdf]

Supplementary Material

1 Supplementary Tables

Table of Contents

Appendix Table 1. Web of Science core collection search formula and search results

Appendix Table 2. PubMed search formula and search results

Appendix Table 3. Embase database search formula and search results

Appendix Table 1. Web of Science core collection search formula and search results

Search Number	Search Query	Results
**1**	((TS=(Pregnancy)) OR TS=(Pregnancies)) OR TS=(Gestation)	447555
**2**	((((((TS=(Pheochromocytoma)) OR TS=(Pheochromocytomas)) OR TS=("Pheochromocytoma, Extra-Adrenal")) OR TS=(Extra-Adrenal Pheochromocytoma)) OR TS=(Extra-Adrenal Pheochromocytomas)) OR TS=("Pheochromocytoma, Extra Adrenal")) OR TS=("Pheochromocytomas, Extra-Adrenal")	14416
**3**	(((((TS=(Paraganglioma)) OR TS=(Paragangliomas)) OR TS=(Paragangliomata)) OR TS=("Paraganglioma, Gangliocytic")) OR TS=(Gangliocytic Paraganglioma)) OR TS=(Gangliocytic Paragangliomas)	7724
**4**	#1 AND (#2 OR #3)	250

Appendix Table 2. PubMed search formula and search results

Search Number	Search Query	Filters	Search Details	Results
**2**	(("Pheochromocytoma"[Mesh]) OR "Paraganglioma"[Mesh]) AND "Pregnancy"[Mesh]		("Pheochromocytoma"[MeSH Terms] OR "Paraganglioma"[MeSH Terms]) AND "Pregnancy"[MeSH Terms]	705
**3**	"Pheochromocytoma"[Title/Abstract] OR "Pheochromocytomas"[Title/Abstrac		"Pheochromocytoma"[Title/Abstract] OR "Pheochromocytomas"[Title/Abstract	20,246

	t] OR "pheochromocytoma extra adrenal"[Title/Abstract] OR "extra adrenal pheochromocytoma"[Title/Abstract] OR "extra adrenal pheochromocytomas"[Title/Abstract] OR "pheochromocytoma extra adrenal"[Title/Abstract] OR "pheochromocytomas extra adrenal"[Title/Abstract]	] OR "pheochromocytoma extra adrenal"[Title/Abstract] OR "extra adrenal pheochromocytoma"[Title/Abstract] OR "extra adrenal pheochromocytomas"[Title/Abstract] OR "pheochromocytoma extra adrenal"[Title/Abstract] OR "pheochromocytomas extra adrenal"[Title/Abstract]	
**5**	"Pregnancy"[Title/Abstract] OR "Pregnancies"[Title/Abstract] OR "Gestation"[Title/Abstract]	"Pregnancy"[Title/Abstract] OR "Pregnancies"[Title/Abstract] OR "Gestation"[Title/Abstract]	621,446
**10**	((("Paraganglioma"[Title/Abstract] OR "Paragangliomas"[Title/Abstract] OR "Paragangliomata"[Title/Abstract]) OR (Paraganglioma, Gangliocytic[Title/Abstract])) OR (Gangliocytic Paraganglioma[Title/Abstract])) OR (Gangliocytic Paragangliomas[Title/Abstract])	"Paraganglioma"[Title/Abstract] OR "Paragangliomas"[Title/Abstract] OR "Paragangliomata"[Title/Abstract] OR (("Paraganglioma"[MeSH Terms] OR "Paraganglioma"[All Fields] OR "Paragangliomas"[All Fields]) AND "Gangliocytic"[Title/Abstract]) OR "gangliocytic paraganglioma"[Title/Abstract] OR "gangliocytic paragangliomas"[Title/Abstract]	9,473
**11**	#3 OR #10 AND #5	("Pheochromocytoma"[Title/Abstract] OR "Pheochromocytomas"[Title/Abstract] OR "pheochromocytoma extra adrenal"[Title/Abstract] OR "extra adrenal pheochromocytoma"[Title/Abstract] OR "extra adrenal pheochromocytomas"[Title/Abstract] OR "pheochromocytoma extra adrenal"[Title/Abstract] OR "pheochromocytomas extra adrenal"[Title/Abstract] OR ("Paraganglioma"[Title/Abstract] OR "Paragangliomas"[Title/Abstract] OR "Paragangliomata"[Title/Abstract] OR (("Paraganglioma"[MeSH Terms] OR "Paraganglioma"[All Fields] OR "Paragangliomas"[All Fields]) AND "Gangliocytic"[Title/Abstract]) OR "gangliocytic paraganglioma"[Title/Abstract] OR "gangliocytic paragangliomas"[Title/Abstract])) AND ("Pregnancy"[Title/Abstract] OR "Pregnancies"[Title/Abstract] OR "Gestation"[Title/Abstract])	460

**12**	#2 OR #11		(("Pheochromocytoma"[MeSH Terms] OR "Paraganglioma"[MeSH Terms]) AND "Pregnancy"[MeSH Terms]) OR (("Pheochromocytoma"[Title/Abstract] OR "Pheochromocytomas"[Title/Abstract] OR "pheochromocytoma extra adrenal"[Title/Abstract] OR "extra adrenal pheochromocytoma"[Title/Abstract] OR "extra adrenal pheochromocytomas"[Title/Abstract] OR "pheochromocytoma extra adrenal"[Title/Abstract] OR "pheochromocytomas extra adrenal"[Title/Abstract] OR ("Paraganglioma"[Title/Abstract] OR "Paragangliomas"[Title/Abstract] OR "Paragangliomata"[Title/Abstract] OR (("Paraganglioma"[MeSH Terms] OR "Paraganglioma"[All Fields] OR "Paragangliomas"[All Fields]) AND "Gangliocytic"[Title/Abstract]) OR "gangliocytic paraganglioma"[Title/Abstract] OR "gangliocytic paragangliomas"[Title/Abstract])) AND ("Pregnancy"[Title/Abstract] OR "Pregnancies"[Title/Abstract] OR "Gestation"[Title/Abstract]))	837
**13**	#2 OR #11	from 1990 - 2024	((("Pheochromocytoma"[MeSH Terms] OR "Paraganglioma"[MeSH Terms]) AND "Pregnancy"[MeSH Terms]) OR (("Pheochromocytoma"[Title/Abstract] OR "Pheochromocytomas"[Title/Abstract] OR "pheochromocytoma extra adrenal"[Title/Abstract] OR "extra adrenal pheochromocytoma"[Title/Abstract] OR "extra adrenal pheochromocytomas"[Title/Abstract] OR "pheochromocytoma extra adrenal"[Title/Abstract] OR "pheochromocytomas extra adrenal"[Title/Abstract] OR ("Paraganglioma"[Title/Abstract] OR "Paragangliomas"[Title/Abstract] OR "Paragangliomata"[Title/Abstract] OR (("Paraganglioma"[MeSH Terms]	491

	OR "Paraganglioma"[All Fields] OR "Paragangliomas"[All Fields]) AND "Gangliocytic"[Title/Abstract]) OR "gangliocytic paraganglioma"[Title/Abstract] OR "gangliocytic paragangliomas"[Title/Abstract])) AND ("Pregnancy"[Title/Abstract] OR "Pregnancies"[Title/Abstract] OR "Gestation"[Title/Abstract]))) AND (1990:2024[pdat])

Appendix Table 3. Embase database search formula and search results

Search Number	Search Query	Results
**#1**	'pregnancy'/exp	921720
**#2**	'pheochromocytoma'/exp	28756
**#3**	'paraganglioma'/exp	16276
**#4**	#2 OR #3	40717
**#5**	#1 AND #4	926
**#6**	'pregnancy':ti,ab,kw OR 'child bearing':ti,ab,kw OR childbearing:ti,ab,kw OR gestation:ti,ab,kw OR gravidity:ti,ab,kw OR 'intrauterine pregnancy':ti,ab,kw OR 'labor presentation':ti,ab,kw OR 'labour presentation':ti,ab,kw OR 'pregnancy maintenance':ti,ab,kw OR 'pregnancy trimesters':ti,ab,kw	803129
**#7**	'pheochromocytoma':ti,ab,kw OR 'chromaffin cell tumor':ti,ab,kw OR 'chromaffin paraganglioma':ti,ab,kw OR phaeochromoblastoma:ti,ab,kw OR phaeochromocytoma:ti,ab,kw OR 'pheochromoblastoma':ti,ab,kw OR pheochromocytomata:ti,ab,kw OR pheochromocytomatosis:ti,ab,kw OR pheochromocytosis:ti,ab,kw	26673
**#8**	paragangliomata:ti,ab,kw OR paragangliomatosis:ti,ab,kw OR 'paraganglion tumor':ti,ab,kw OR 'paraganglion tumour':ti,ab,kw OR paraganglioneuroma:ti,ab,kw OR 'paraganglionic tumor':ti,ab,kw OR paraganglioma:ti,ab,kw	10412
**#9**	#7 OR #8	33094
**#10**	#6 AND #9	738
**#11**	#5 OR #10	1108
**#20**	#11 AND (1990:py OR 1991:py OR 1992:py OR 1993:py OR 1994:py OR 1995:py OR 1996:py OR 1997:py OR 1998:py OR 1999:py OR 2000:py OR 2001:py OR 2002:py OR 2003:py OR 2004:py OR 2005:py OR 2006:py OR 2007:py OR 2008:py OR 2009:py OR 2010:py OR 2011:py OR 2012:py OR 2013:py OR 2014:py OR 2015:py OR 2016:py OR 2017:py OR 2018:py OR	792

	2019:py OR 2020:py OR 2021:py OR 2022:py OR 2023:py OR 2024:py)
